# Chitosan Biopolymer from Crab Shell as Recyclable Film to Remove/Recover in Batch Ketoprofen from Water: Understanding the Factors Affecting the Adsorption Process

**DOI:** 10.3390/ma12233810

**Published:** 2019-11-20

**Authors:** Vito Rizzi, Jennifer Gubitosa, Paola Fini, Roberto Romita, Sergio Nuzzo, Pinalysa Cosma

**Affiliations:** 1 Dipartimento di Chimica, Università degli Studi “Aldo Moro”, Bari, 4-70126 Bari, Italy; vito.rizzi@uniba.it (V.R.); roberto.romita@uniba.it (R.R.); 2 Consiglio Nazionale delle Ricerche CNR-IPCF, UOS Bari, 4-70126 Bari, Italy; j.gubitosa@ba.ipcf.cnr.it (J.G.); p.fini@ba.ipcf.cnr.it (P.F.); sergio.nuzzo@ba.ipcf.cnr.it (S.N.)

**Keywords:** chitosan film, emerging pollutants, ketoprofen, food waste, adsorption, recycle

## Abstract

Seafood, a delight for many people, is sold in the market as a wide variety of products. However, seafood industries produce many by-products; for example, during the processing, the heads and shells of shellfish are generated as waste. This results in the generation of a large amount of shell waste that is accumulated over time, inducing a major environmental concern. Effective solutions for recycling shell waste should be taken into consideration, and the extraction of commercially useful substances like chitin and its derivates, such as chitosan, could be a valid solution for reducing the seafood waste’s environmental impact. Thus, during this work, we propose the use of chitosan as biowaste, to induce the formation of solid films useful for decontaminating water from emerging pollutants. In particular, ketoprofen was used as a model contaminant, and a high percentage of removal, at least 90%, was obtained in a short time under our experimental conditions. Thus, a comprehensive investigation into the adsorption of ketoprofen onto chitosan film was performed, detailing the nature of the adsorption by studying the effects of pH, temperature changes, and electrolyte presence in the solutions containing the pollutant. The process was found to be pH-dependent, involving meanly electrostatic interactions between the pollutant molecules and chitosan. The endothermic character of the adsorption was inferred. The kinetics of the process was investigated, showing that the pseudo second-order kinetic model best fit the experimental data. A recycling process of the adsorbent was proposed; therefore, the adsorbed pollutant can be recovered by reusing the same adsorbent material for further consecutive cycles of adsorption without affecting the efficiency for ketoprofen removal from water.

## 1. Introduction

The production of shell waste by the seafood industry is, among other environmental problems, one of noteworthy concern, contributing to environmental and health hazards [1]. Indeed, a great amount of crab and shrimp shells are produced as waste by worldwide seafood companies [2]. Unfortunately, about 45% of waste resulting from processed seafood is disposed as landfill, consequently leading to environmental pollution in terms of odor and aesthetic damage to the environment [3]. To dispose of this waste, burning is proposed; however, this solution is costly due to the low burning capacity of shells [1]. 

However, it is worth mentioning that fishery by-products have economical value for chitin and chitosan production [3]; thus, the conversion of shell waste to commercial products could be considered an effective approach for shell waste remediation [1]. Accordingly, Premasudha et al. [4] reported that the ocean ecosystem is one of the major sources of biopolymers, particularly chitosan, increasing the interest toward this bio-waste material, which is characterized by very interesting applications due to its chemical structure and properties [4]. Indeed, chitosan, a by-product from the alkaline deacetylation process of chitin [5], is an amino polysaccharide known for its distinctive properties, as well as its biodegradability and biocompatibility [4]. 

Chitosan has great potential for environmental applications [6], such as the remediation of organic and inorganic contaminants, including toxic metals and dyes in soil, sediment, and water [7,8,9,10], and for the development of devices [11,12]. Among pollutants [13,14], emerging contaminants, e.g., pesticides and their metabolites, pharmaceuticals, personal and house care products, life-style compounds, food additives, industrial products and waste, and nanomaterials, are a great and important problem for the environment. The cumulative use of these substances led to their relatively recent appearance in detectable levels in soils, as well as surface and groundwater resources, with unpredictable consequences for ecosystems [15]. The removal of these pollutants from the environment must be taken into account, and the development of a more sustainable and greener technology for this purpose should be developed [16]. Addressing this concern, several studies [17,18] were reported in the literature, with the use of adsorption methods suggested as the most powerful tools. [18] Furthermore, in order to reduce the environmental impact and the associated cost, agricultural and food waste was carefully investigated as a tool for the removal of pollutants [19,20]. Starting from these considerations, with the aim of valorizing food waste, particularly chitosan from crab shell, attention in this work was focused on the removal of ketoprofen (Kp), adopted as a model emerging pollutant. The use of chitosan as a solid film (CH) is proposed, exhibiting enormous advantages in the treatment of water for Kp removal. Kp, a non-steroidal anti-inflammatory drug (NSAID), is frequently found in surface water, constituting a potential risk for aquatic ecosystems [21,22,23]. 

Indeed, for its removal, Jankunaite et al. [24] proposed the use of advanced oxidation processes (AOPs). In this regard, however, it is worth mentioning that AOPs suffer from side effects due to the potential toxicity of the induced by-products, which are often more toxic than the parent molecules [24]. 

Nagy et al. [25] reported the use of cyclodextrin-based polymers, working in synergy with filters, to exploit the possibility of working under dynamic conditions [25]. Madikizela et al. [26] showed the efficiency of molecularly imprinted polymers, designed for the selective extraction of ketoprofen from wastewater [26]. 

In addition to these studies, other proposed uses of CH to treat water for removal of the emerging contaminant Kp were outlined in our previous paper devoted to the removal of diclofenac [27]. In the present work, a deep investigation into the process is presented. The use of chitosan film from food waste should not only lower the associated adsorption processes, if compared with the previous adsorbents, but also improve the environmental impact of seafood waste, valorizing it with a simple and easy method to obtain materials for treating water with Emerging Contaminants (EC). Moreover, during this work, for the first time, in addition to the possibility of decontaminating water containing Kp, we propose the recovery of CH after adsorption, emphasizing the sustainable character of the described approach. Indeed, 10 consecutive cycles of adsorption/desorption were performed without affecting Kp removal and recovery, which were completed in a few minutes.

## 2. Materials and Methods

### 2.1. Chemicals

The used chemicals were of analytical grade, and the samples were prepared using deionized water. Highly viscous chitosan powder, from crab shells (with a molecular weight MW of 150,000 and a deacetylation degree ≥75%, Sigma-Aldrich, Milan, Italy) acetic acid (99.9% Sigma-Aldrich, Milan, Italy), and glycerol (+99.5%, Sigma-Aldrich, Milan, Italy) were obtained from Sigma-Aldrich. The same commercial source was adopted for sodium ketoprofen (C_16_H_13_NaO_3_, MW: 276.267 g∙mol^−1^ Sigma-Aldrich, Milan, Italy), used without further purification. A Kp stock solution with a concentration of 5 mg∙L^−1^ was prepared. The pH of the various aqueous solutions, when necessary, was adjusted using concentrated HCl and NaOH solutions. Dilutions were performed using deionized water. 

### 2.2. Preparation of Chitosan Films

Chitosan powder was dissolved in a 0.8% (*v*/*v*) aqueous acetic acid solution to obtain a 1% (*w*/*v*) chitosan concentration by continuous stirring for 24 h. Then, 200 μL of glycerol was added to 100 mL of the chitosan/acetic acid solution. The solution was degassed for 1 h and poured into a plastic Petri plate, which was maintained in an oven at 60 °C for 24 h, thereby obtaining CH. The thickness of the obtained film was about 1 mm.

### 2.3. UV–Visible Light (UV–Vis) Measurements

UV-Vis spectra were recorded using a Varian CARY 5 UV–Vis/near-infrared (NIR) spectrophotometer (Varian Inc., now Agilent Technologies Inc., Santa Clara, CA, USA). Spectra were recorded in the 200–800 nm range, with a 1 nm/s scan rate, and the Kp amounts were monitored by measuring the absorbance intensity at λ = 260 nm. The molar absorption coefficient (ε) of 13,530 L∙mol^−1^∙cm^−1^, experimentally inferred, was used to obtain the Kp concentration in water. All experiments were performed in triplicate, calculating the relative standard deviations.

### 2.4. In Batch Equilibrium Experiments

The CH adsorption capacities were calculated as q_t_ (mg∙g^−1^) at different contact times (t), by applying Equation (1) [13,14,16].

(1) $$q_{t}=\frac{C_{0}-C_{t}}{W}\times V,$$

where V represents the Kp solution volume (herein 15 mL), W is the dried chitosan adsorbent mass (g), and C_0_ and C_t_ (mg∙L^−1^) represent the initial Kp concentration and the Kp concentration at time t. 

A fixed amount of CH (150 mg) was added to flasks containing 15 mL of Kp solution at two initial concentrations (1 × 10^−5^ M and 5 × 10^−6^ M, corresponding to ~2.5 mg∙L^−1^ and ~1.25 mg∙L^−1^, respectively). The adsorption was performed under continuous stirring at 250 rpm, and UV–Vis absorption spectra were recorded to evaluate the Kp removal efficiency and the chitosan adsorption capacities from water. Furthermore, the effect of adsorbent amount was also explored, changing its mass from 35 mg to 200 mg. In this case, the Kp concentration was maintained at 1 × 10^−5^ M. The effects of solution ionic strength and changes in pH values (ranging from 3 to 12) on the adsorption process were also studied. 

### 2.5. In Batch Desorption Experiments

After the Kp adsorption from water, NaCl (0.25 M) was selected, with regard to a green economy and cleaner production, as the best salt to induce the release of the adsorbed Kp. With the same approach adopted for Kp adsorption, the UV–Vis investigation was used to assess the amount of desorbed pollutant. After the Kp adsorption, the adsorbent was washed with fresh water to remove the non-adsorbed Kp swollen in the NaCl solution for release. The effect of the contact time was evaluated, and 30 min was found to be suitable for Kp recovery. 

### 2.6. Adsorption Kinetics

Information about the kinetics of the adsorption process between Kp and the chitosan film was inferred by adopting both pseudo first-order and pseudo second-order kinetic models. The following linearized equations for the pseudo first-order (Equation (2)) and pseudo second-order (Equation (3)) models were adopted [13,14,16]:

(2) $$\ln\left(q_{e}-q_{t}\right)=\ln\left(q_{e}\right)-K_{1}\times \;t,$$

(3) $$\frac{t}{q_{t}}=\frac{1}{K_{2}q_{e}^{2}}+\frac{1}{q_{e}}\times \;t,$$

where q_e_ and q_t_ represent the adsorption capacities at equilibrium and at time t, respectively (mg∙g^−1^), and k_1_ (min^−1^) and k_2_ (g∙mg^−1^∙min^−1^) are the rate constants of the pseudo first-order and second-order models, respectively. 

### 2.7. Thermodynamic Studies

Free energy (ΔG°), entropy (ΔS°), and enthalpy (ΔH°) were calculated [27] for the Kp adsorption onto the chitosan film at three selected temperatures of 298, 288, and 278 K. The value of ΔG° was inferred using Equation (4).

ΔG° = −RT *ln* K_eq_, (4)

where *R* is the universal gas constant (8.314 J/mol∙K), *T* is the temperature (K), and K_eq_ represents the equilibrium constant that was calculated as q_e_/C_e_, where q_e_ is the sorption capacity (mg∙g^−1^) at equilibrium, and C_e_ is the equilibrium concentration (mg∙g^−1^). The values of Δ*H°* and Δ*S°* were determined by combining Equation (4) with Equation (5), thereby obtaining Equation (6). 

ΔG° = ΔH° − TΔS°.(5)

(6) $$\ln K{}_{\mathrm{eq}}=-\frac{\Delta H{}^{\circ}}{RT}+\frac{\Delta S{}^{\circ}}{R}.$$

### 2.8. Determination of Chitosan Film Zero-Point Charge

The zero-point charge pH (pH_ZPC_) of the chitosan adsorbent was evaluated by using the pH drift method [14]. Firstly, 30 mL of NaCl solution with a concentration of 5.0 × 10^−2^ M was used at different pH values ranging from 2 to 12 (pH_i_). Concentrated HCl and NaOH solutions were used for this purpose. The pH_i_ values of these solutions were measured, and 25 mg of adsorbent was subsequently introduced. These solutions were stirred at 298 K for 48 h. The final pH (pH_F_) values were measured. By reporting the pH_i_ versus pH_F_ values, the value of pH_ZPC_ was inferred at the cross-section of the latter curve with the line of pH_i_ versus pH_i_. All experiments were performed in triplicate, calculating the relative standard deviations.

## 3. Results and Discussion

**Figure 1:**
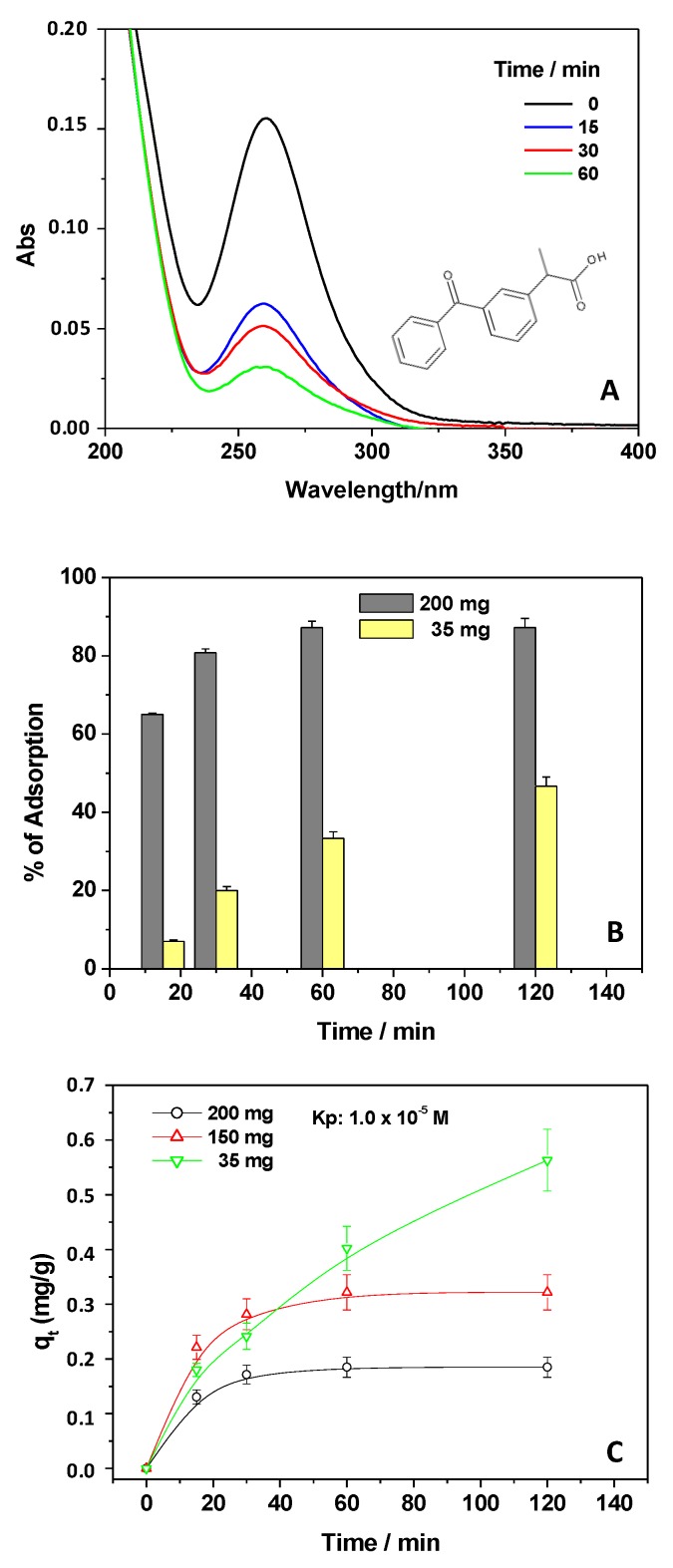
Ultraviolet–visible light (UV–Vis) spectra of a 1 × 10^−5^ M ketoprofen (Kp) solution, pH 5, collected at different contact times, in the presence of 150 mg of adsorbent (**A**); Kp adsorption percentage (1 × 10^−5^ M, pH 5) calculated at different contact times in the presence of 200 mg and 35 mg of adsorbent (**B**); adsorption capacities, qt, referring to different amounts of chitosan (200, 150, and 35 mg) in contact with a 1 × 10^−5^ M Kp solution, pH 5 (**C**).

The UV–Vis absorption spectrum of Kp was used to monitor its removal from contaminated water. As a first step, an aqueous solution purposely spiked with the drug was investigated, and the Kp spectroscopic main signal at 260 nm (Figure 1A) was followed.

By adopting 150 mg of adsorbent, in the presence of 1 × 10^−5^ M Kp, the adsorption was followed until 60 min. The main process of Kp removal was observed in the first 15 min, exhibiting an efficiency of 55%; by extending the contact time, the effect resulted less pronounced with respect to the beginning of the process, and, in 60 min, almost 85% of the NSAID was eliminated from the water (Figure 1A). The results suggested the probable key role of the Kp concentration gradient (ΔC) between the bulk of the solution and the adsorbent surface [28]. At the beginning of the adsorption process, the ΔC was high enough to induce the diffusion of the NSAID from the bulk of the solution at the surface of the adsorbent; on the other hand, upon extending the contact time, the ΔC was reduced, thereby slowing down the Kp adsorption. Furthermore, at the beginning of the process, the presence of a large number of free sites on the adsorbent surface for Kp adsorption also favored the NSAID removal. However, upon extending the contact time, the number of available free sites to host Kp decreased, reducing the relative Kp adsorption (Figure 1A) [13,14,16,29].

Moreover, this behavior could be attributed to the presence of repulsive forces between free Kp molecules in solution and those adsorbed, which further hindered the drug adsorption [30].

After these assessments, the effects of several parameters affecting the NSAID removal from water were investigated, while also evaluating the effects on the adsorption capacities. 

### 3.1. Effects of Adsorbent Dosage and Kp Concentration

To study the effect of the CH amount on the Kp removal from water, thus evidencing the role of active sites hosting Kp, two other CH films with different weights (200 and 35 mg) were compared under the same experimental conditions (Figure 1B), i.e., a 1 × 10^−5^ M Kp solution at pH 5 (pH of the Kp solution soon after adsorbent addition). 

The Kp removal efficiencies were calculated, and adsorption percentages of 65% and 8% were determined in the first 15 min when using the greatest and the smallest amounts of adsorbent, respectively; as shown in Figure 1B, these efficiencies were increased upon extending the contact time to 120 min, obtaining 90% Kp removal if in presence of the largest amount of CH. 

This result confirmed the previously mentioned importance of free active sites able to host the NSAID [13,14,16,29], which increase in number upon increasing the available surface of the adsorbent. Overall, in Figure 1C, the influence of the chitosan amount on Kp adsorption was evaluated by reporting the q_t_ values (Equation (1)) as a function of the three investigated CH weights. 

The obtained results indicated that, by increasing the adsorbent amount, the relative adsorption of Kp molecules increased (see the plateaus on the graph), while the adsorption capacity decreased. This suggests that, despite the great efficacy, by using a large amount of adsorbent, the adsorption sites remained partially unsaturated during the sorption process, reducing the q_t_ values as a whole [13,14]. In particular, as already observed by monitoring the Kp UV–Vis absorption spectrum (Figure 1A), at the beginning of the adsorption process (Figure 1C), the presence of a large quantity of free sites for Kp adsorption and the high ΔC increased the q_t_ values. On the contrary, by extending the contact time, the free sites and ΔC decreased, reducing the Kp adsorption overall, leading to a plateau. All of these findings agree with the results obtained upon varying the Kp concentrations as described below. 

**Figure 2:**
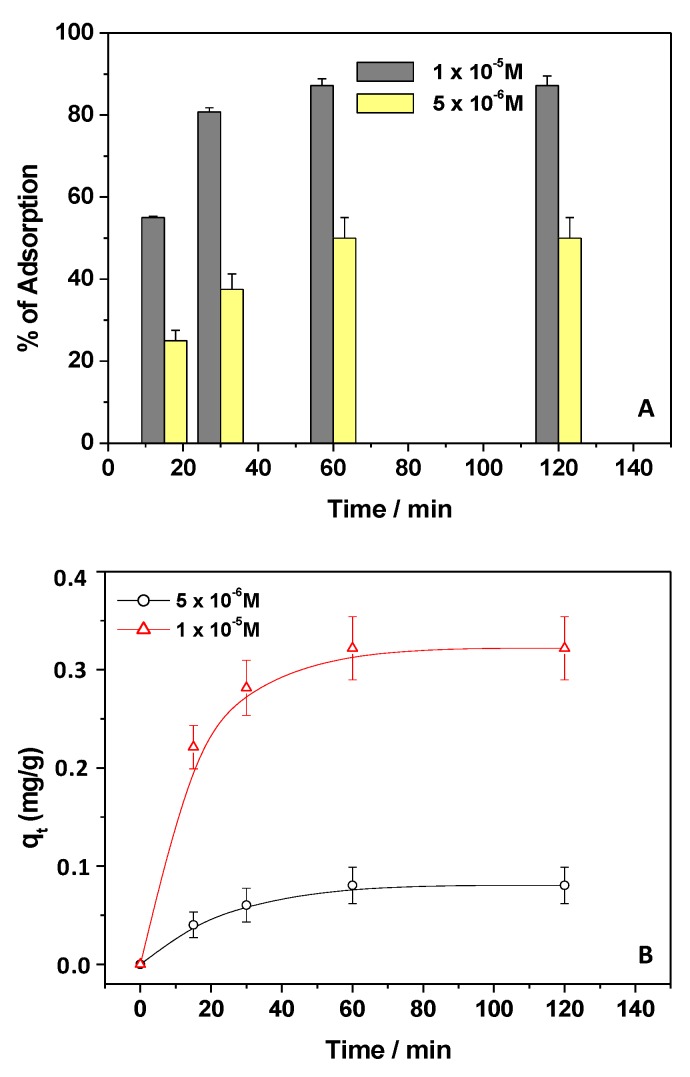
Percentage of Kp adsorption (**A**) and adsorption capacities, qt (**B**), referring to different Kp concentrations of 1 × 10^−5^ M and 5 × 10^−6^ M, pH 5, in the presence of 150 mg of adsorbent.

For this purpose, the concentration of Kp was changed (1 × 10^−5^ M and 5 × 10^−6^ M), fixing the chitosan weight at 150 mg, as shown in Figure 2A. In both cases, the Kp removal showed great variation in the first minute of contact, showing that, upon diluting the Kp solution, the pollutant removal percentage decreased. Not surprisingly, the dilution of Kp solutions (with a dilution factor of 1:2) reduced the percentage of Kp removal from 65% to 25% in the first 15 min and from 85% to 50% after 120 min. Accordingly, the associated q_t_ values were calculated, as reported in Figure 2B, showing higher q_t_ values for the concentrated Kp solution. Moreover, in the latter case, the relative maximum adsorption capacities were quickly obtained after a few min, as compared with the diluted Kp solution.

### 3.2. Kinetic Analysis

**Figure 3:**
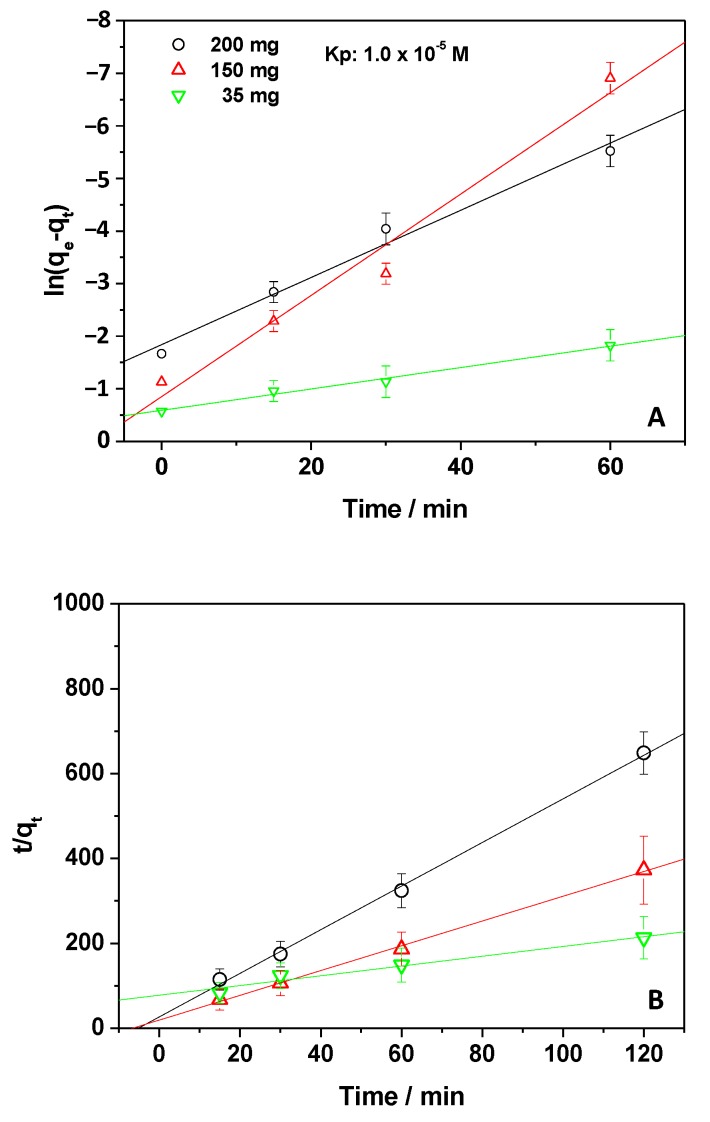
Pseudo first-order (**A**) and second-order (**B**) kinetic models applied to experiments of adsorption in which the amount of the adsorbent was changed.

Information about the dynamics of the adsorption was obtained by investigating the kinetics of the process, applying pseudo first-order and pseudo second-order kinetic models (Equations (2) and (3)). By using the *q_t_* values reported in Figure 1B, the results shown in Figure 3A,B were obtained. Table 1 reports the calculated kinetic parameters.

The best kinetic model to describe the experimental data was evaluated by comparing the *R^2^* values of the linear fitting, as well as the experimental adsorption capacities at equilibrium, q_e,exp_ (contact time 120 min), with those obtained by applying the kinetic equations, q_e,calc_ (calculated adsorption capacities) [13,14]. From Table 1, the *R^2^* values and the comparison between q_e,exp_ and q_e,calc_ suggest that the application of the pseudo second-order equation better described the experimental data, emphasizing the role of both Kp and chitosan amounts during the adsorption process [13,14,16]. Antunes et al. [31] reported that the use of this kinetic model indicates that the rate-controlling step depends on both physical and chemical interactions between the pollutant and adsorbent [31]. However, it is worth pointing out that, from the data reported in Table 1, when using the smallest CH amount, the pseudo first-order model is probably preferred. This suggests that, under this condition, the rate limiting step could be the Kp concentration, i.e., the diffusion of Kp mainly controls the removal of the NSAID [28]. 

**Figure 4:**
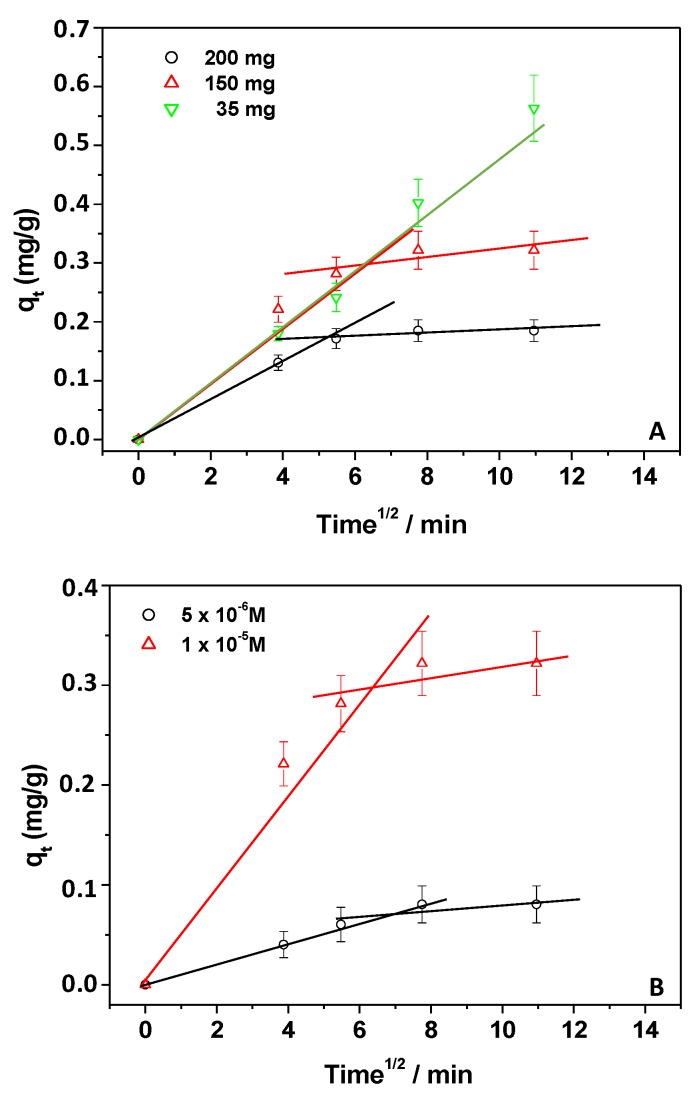
Weber–Morris plot applied to experiments of adsorption in which the amounts of the adsorbent (**A**) and Kp (**B**) were changed.

Additional information was obtained by adopting the Weber–Morris model (W–M). The intra-particle diffusion model was used, as described by the following equation: q_t_ = k_int_ × t^1/2^ + C, where *C* represents the thickness of the boundary layer, and k_int_ is the kinetic constant related to the intra-particle diffusion rate in mg∙g^−1^∙min^−1/2^ [32]. As reported by Lin et al. [32], if the plot of q_t_ versus t^1/2^ is represented by a straight line passing through the origin, the intra-particle diffusion is the limiting stage of the adsorption. On the other hand, if multiple linear segments are necessary to fit the experimental data, two or more steps could be involved during the NSAID adsorption process [31,32]. The W–M equation was, thus, applied (Figure 4A,B) to the q_t_ values reported in Figure 1C and Figure 2B, referring to experiments in which the amounts of both CH and Kp were changed.

As a whole, the findings suggested that, under our experimental conditions, the Kp adsorption process could be described by two steps: (i) diffusion from the solution to the external surface of the adsorbent, and (ii) intra-particle adsorption and diffusion. Indeed, the experimental points could be divided into these two stages (Figure 4A,B). Moreover, during the second stage, since the ΔC of Kp decreased, the adsorption process decreased, reaching an equilibrium state. Once again, the exception was represented by the condition in which the smallest amount of chitosan was used. In this case, the regression line of the first stage passed through the origin of the plot, suggesting that the intra-particle diffusion was the rate-limiting step [31]. In this latter case, the number of available free sites present on the CH surface was lowered, and the diffusion controlled the process as previously supposed. 

### 3.3. Effect of pH

To get insight into the nature of the adsorption process, the effect of pH during the adsorption of Kp onto CH was investigated, by adding either HCl or NaOH. To avoid changes in pH values during the adsorption process, due to the presence of acetic acid added during chitosan film preparation, the used adsorbent (150 mg) was neutralized with NaOH and washed several times with fresh water, until a neutral pH was achieved. 

**Figure 5:**
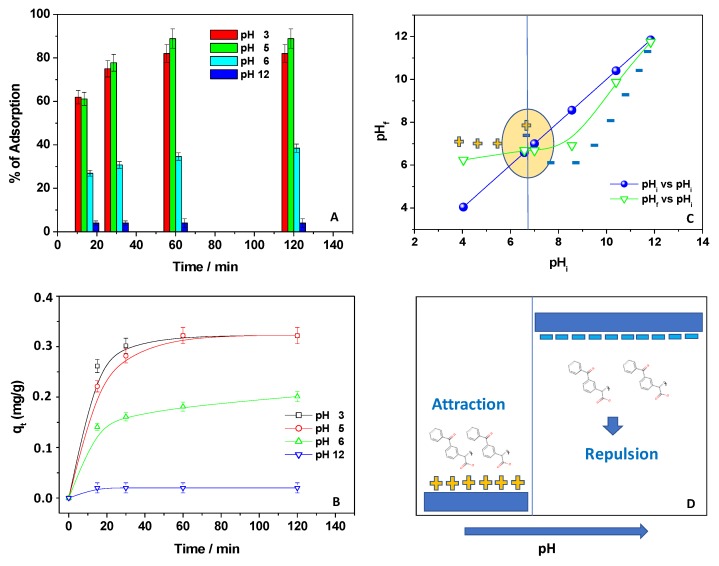
Percentage of Kp adsorption from an aqueous solution (1 × 10^−5^ M) when 150 mg of chitosan was used at different pH values (**A**); drift method to determine the zero-point charge of the adsorbent (**B**); adsorption capacities, q_t_, referring to Kp adsorption from an aqueous solution (1 × 10^−5^ M) when 150 mg of chitosan was used at different pH values (**C**); cartoon depicting the interaction between Kp and the adsorbent (**D**).

As reported in Figure 5A, the percentage of Kp adsorption was calculated at each pH value and contact time. The maximum adsorption occurred between pH 3 and pH 5, while it decreased after this pH value, with the lowest Kp removal at pH 12. Interestingly, at the beginning of the process, i.e., in the first 15 min, the Kp removal was approximately the same at pH 3 and 5, and it was reduced upon increasing the pH value. Instead, upon extending the contact time, the affinity at pH 3 was slightly reduced, and the results at pH 5 appeared the best. These findings were better evidenced by calculating the associated q_t_ values (Figure 5B). 

Clearly, at both pH 3 and pH 5, the adsorption capacities were good, having the highest values, and, at pH 3, the q_t,max_ was reached quickly in the first minute. In order to better understand this behavior, the zero-point charge (ZPC) of CH was determined using the drift method [14] (Figure 5C). The observed cross-section region of the curves in Figure 5C indicated that the pH_ZPC_ of CH was around pH 7. This means that, below pH 7, the chitosan amino groups were positively charged, while they were deprotonated toward the pH_ZPC_. After this pH value, chitosan became mainly negatively charged [33]. The negative charge on the surface of the chitosan film in alkaline medium could be mainly ascribed to the presence of negative ions (OH^−^) in solution, that would form a negative layer on the surface of chitosan. Moreover, in accordance with the carboxylic moieties present in the Kp chemical structure (see Figure 1A), the NSAID pK_a_ is reported to be around 4 [34]. This means that, below this pH value, Kp was present as a neutral molecule (Kp-H), while, above this value, it was present as an anionic one (Kp^−^). 

Thus, below pH 5, Kp was present as Kp-H, and CH showed a positively charged surface. As a result, a reduced affinity between Kp-H and the adsorbent was expected due to the reduced contribution of electrostatic interactions. However, since adsorption at pH 3 was quite significant, other forces should probably be considered during the process, such as dipole–dipole interactions and H-bonds. At pH 5, Kp was mainly present as Kp^−^ and, at the same time, chitosan was positively charged; thus, electrostatic interactions between the carboxylic moieties of Kp^−^ and the chitosan’s positively charged amino groups took place, favoring pollutant removal. At pH > 5, i.e., pH 6, the chitosan amino groups were mainly deprotonated, thereby reducing the affinity between the adsorbent and the NSAID, thus suggesting an electrostatic repulsion between the negative Kp and CH charges [35,36,37,38]. This effect was more evident at pH 12, at which the adsorption was completely blocked. Figure 5D reports the possible scheme of interaction between Kp and CH, considering mainly electrostatic forces. 

### 3.4. Effect of Salts in Kp Solutions

With the aim of assessing the role of the electrostatic interaction between the Kp^−^ anion and chitosan, some experiments were performed, changing the ionic strength of Kp solutions by adding electrolytes. By selecting NaCl as a model electrolyte at different concentrations, the experiments were performed using 150 mg of chitosan and 1 × 10^−5^ M Kp.

**Figure 6:**
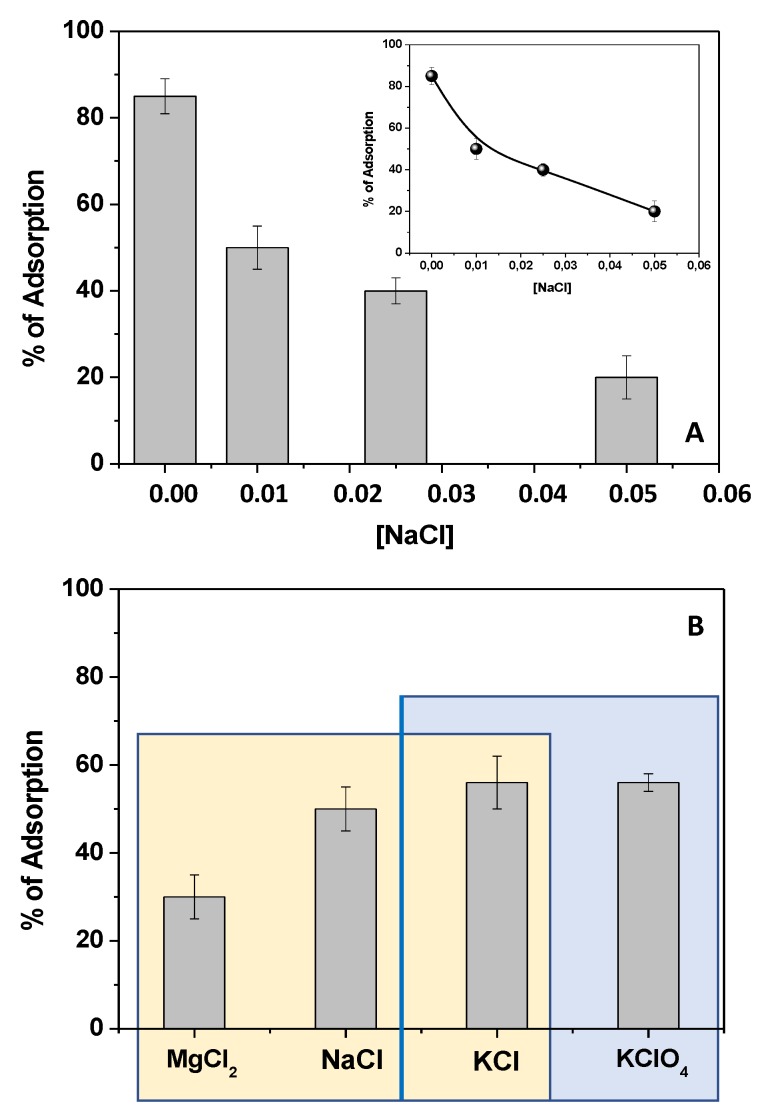
Percentage of Kp adsorption from an aqueous solution (1 × 10^−5^ M, pH 5) when 150 mg of chitosan was used at different concentrations of NaCl (**A**) and in the presence of different salts at 0.01 M (**B**).

Thus, the Kp adsorption was evaluated, and the results obtained at 60 min of contact time are reported in Figure 6A. By changing the salt concentration from 0.01 M to 0.05 M, Kp removal decreased from 85%, in the absence of salt, to 20% with 0.05 M NaCl (Figure 6A). By choosing 0.01 M as the salt reference concentration, the electrolyte nature was changed. In particular, upon fixing the type of anion (Cl^−^), the cation was changed by exploring the effects of Na^+^, K^+^, and Mg^2+^. As reported in Figure 6B, upon decreasing the cation size from K^+^ to Na^+^, the Kp removal efficiency decreased and, upon changing the cation associated charge, using Mg^2+^, the effect became more pronounced.

These results suggested a cation-mediated shielding effect of the Kp negative charge, confirming the involvement of the Kp carboxylic moieties in its adsorption onto the chitosan film. Interestingly, by changing the type of anion, and choosing K^+^ as the cation, the results reported in Figure 6B were obtained. The absence of significant changes in the removal of Kp indicated that the inorganic anions did not affect the adsorption. In fact, in general, if the anion affected the process, its effect would involve the shielding of CH positive charges onto the film surface, slowing down the Kp diffusion into the film, thus preventing adsorption. Instead, the cation effects occurred in solution, shielding the Kp negative charge, thereby reducing the adsorbate/adsorbent affinity. 

### 3.5. Thermodynamic Analysis

**Figure 7:**
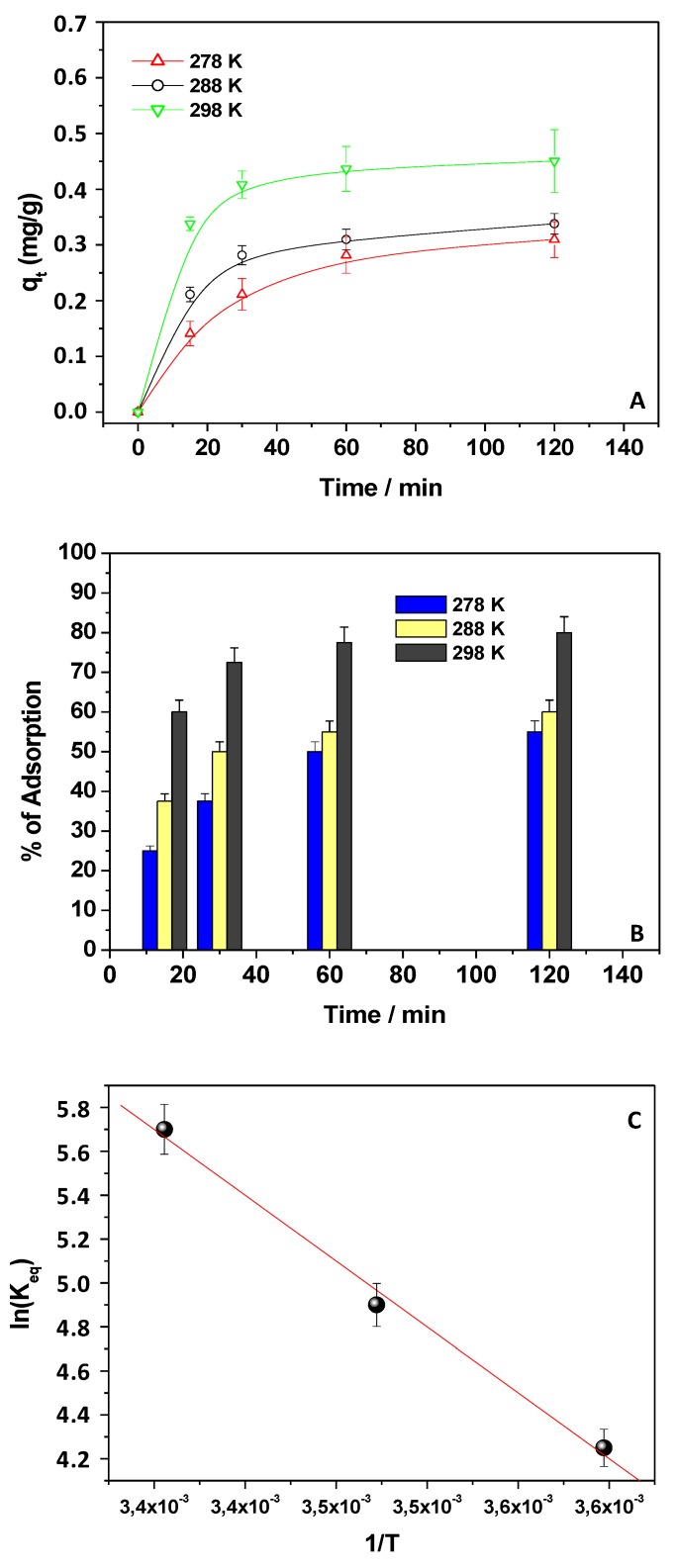
Adsorption capacities, q_t_ (**A**), and percentage of Kp adsorption (**B**) at different contact times, referring to a Kp aqueous solution (1 × 10^−5^ M, pH 5) when 150 mg of chitosan was used at different temperature values; plot of ln (K_eq_) versus 1/T (**C**).

The Kp adsorption process was investigated by adopting three temperature values, i.e., 278, 288, and 298 K, using 150 mg of chitosan and a 1 × 10^−5^ M Kp solution at pH 5. Figure 7 shows the obtained results in term of adsorption capacities (Figure 7A) and percentage of Kp adsorption (Figure 7B) at different contact times. Upon increasing the temperature values, the q_t_ values and the Kp adsorption percentage at the equilibrium increased, indicating the endothermic character of the process. With the aim of obtaining the thermodynamic parameters, the K_eq_ values were calculated at each temperature and, by using Equations (4) and (5), the correspondent ΔG° values were inferred (Table 2). The negative ΔG° values indicated the spontaneity of the Kp adsorption process onto chitosan. Furthermore, by plotting ln K_eq_ versus 1/T (Figure 7C) and applying Equation (6), ΔH° and ΔS° were also calculated (Table 2). In agreement with the literature [31], the positive values of ΔH° and ΔS° confirmed the endothermic character of the process and the increased randomness at the adsorbent–adsorbate interface, respectively.

### 3.6. Consecutive Cycles of Adsorption

With regard to a circular economy and sustainable application, the great performance of the proposed adsorbent was shown upon performing consecutive cycles of adsorption. By selecting 120 min as the contact time, a 1 × 10^−5^ M Kp solution at pH 5 was placed in contact with the adsorbent (150 mg) and, after almost complete adsorption (80% of Kp), the same film was used again to adsorb Kp from another solution at the same concentration. The Kp adsorption percentages reported in Table 3 were obtained. The results indicated that, after eight cycles of adsorption, the NSAID removal was still high, suggesting the possibility of using the same film several times for the removal of the NSAID, thus extending the adsorbent’s lifetime. 

### 3.7. Release of Kp and Reuse of the Adsorbent

**Figure 8:**
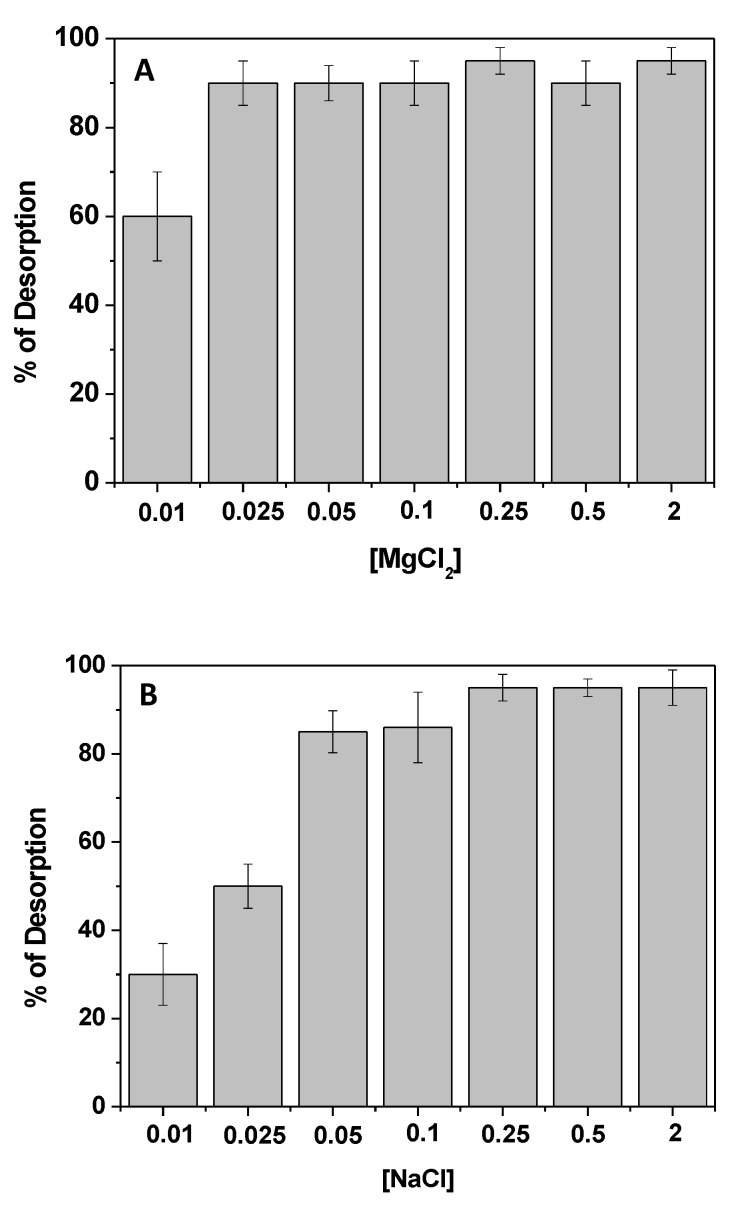
Percentage of Kp desorption, calculated by adopting 30 min as the contact time, in the presence of different concentrations of MgCl_2_ (**A**) and NaCl (**B**).

Another positive aspect of the proposed adsorbent was the possibility to recover the adsorbed pollutant, recycling both the molecule and the adsorbent itself. Indeed, due to the involvement of electrostatic interactions between Kp and chitosan, solutions containing salts were successfully used to obtain the desorption, thus reducing the environmental impact. Among the studied salts (see Figure 8), NaCl and MgCl_2_ solutions were selected and compared. After Kp adsorption, with a contact time of 120 min, the chitosan film was placed in contact with aqueous solutions of NaCl and MgCl_2_ at several concentrations, under continuous stirring. Once again, the Kp UV–Vis absorption spectrum, collected at 30 min, was used to monitor the Kp release. The percentage of the desorbed Kp, normalized for the adsorbed amount, was calculated, and the results are reported in Figure 8. The use of MgCl_2_ ensured the total release of adsorbed Kp, upon using diluted solutions of salts (Figure 8A); on the other hand, the use of NaCl required a larger amount of salt. In fact, Kp release was only reasonable beyond a concentration of 0.25 M NaCl (Figure 8B). 

However, it is worth pointing out that the use of MgCl_2_ induced the slight degradation of the chitosan film. Thus, with a view of a more sustainable procedure for cleaner production and pollution prevention, the use of NaCl 0.25 M is suggested for Kp recovery, applying 30 min as the contact time for its desorption. Several cycles of adsorption/desorption were performed, and the percentage of the adsorbed/desorbed Kp for each cycle is reported in Table 4. Once again, the desorbed Kp percentage was normalized with respect to the correspondent adsorbed Kp. After each cycle, the adsorbent was washed with fresh water, and subsequently placed in contact with a fresh Kp solution. Interestingly, despite the same film being used several times, the efficiencies of Kp removal and recovery were not affected, and the obtained results suggested the possibility of reducing the procedure-associated costs and the amount of secondary pollutants potentially released into the environment, suggesting not only possible Kp reuse, but also adsorbent recycling. 

## 4. Conclusions

Due to concerns related to water decontamination from pollutants, this paper reported the use of chitosan films for the removal of Kp from water, with regard to more ustainable and greener industrial applications. The bio-sorption process is very interesting, since about 90% of the Kp was removed in 2 h at most. The sorption process was investigated at several temperature values, indicating that, upon increasing the temperature, the removal of Kp from water increased. The thermodynamic parameters were calculated, observing that the process was spontaneous (ΔG° < 0) and endothermic (ΔH° < 0), and that it occurred with an increase in entropy. From a kinetic point of view, the pseudo second-order kinetic model agreed well with the experimental data, indicating that the bio-sorption was dependent on the amounts of Kp and adsorbent. In fact, upon increasing the amount of CH and the concentration of the Kp solution, the pollutant removal efficiencies affected the adsorption capacities. The high affinity between the NSAID and CH was ascribed to the presence of electrostatic interactions. Indeed, the adsorption was largely affected by the pH value of the Kp solution and by the presence of salts. 

Considering that the pK_a_ of Kp is about 4, the maximum removal was observed at pH 5, while, upon further increasing the pH values of the Kp solution, the NSAID removal decreased. In accordance with the measured pH_ZPC_ of the CH (~7), below this pH value, the interaction between Kp^−^ and CH was observed. However, below the pK_a_ of the Kp, the main form Kp-H reduced the affinity toward the adsorbent. The same finding was observed at basic pH values, conditions in which the adsorbent was negatively charged and repulsions between Kp^−^ and the adsorbent occurred. An interaction between the Kp carboxylic moiety and the chitosan amino groups was, thus, proposed and confirmed by experiments of adsorption performed in the presence of salts, which inhibited the Kp removal. Interestingly, the use of 0.25 M NaCl was found to be suitable for the desorption of the adsorbed Kp, enabling the reuse of the pollutant and the recycling of the adsorbent for several cycles, extending the CH lifetime. The reuse of the recovered pollutants and the use of CH, proposed in this work, exhibit great benefits for the environment through the reuse of products for cleaner production technologies. 

## Figures and Tables

**Table 1:** Kinetic parameters obtained by applying the pseudo first-order and second-order kinetic models.

Pseudo First-Order					Pseudo Second-Order			
Adsorbent (mg)	q_e(the)_	q_e(exp)_	K_1_	*R^2^*	q_e(the)_	q_e(exp)_	K_2_	*R^2^*
200	0.16	0.18	6.4 × 10^−2^	0.983	0.19	0.18	1.05	0.998
150	0.43	0.32	9.6 × 10^−2^	0.975	0.33	0.32	0.5	0.998
35	0.55	0.56	2.0 × 10^−2^	0.989	0.68	0.56	1.0 × 10^−2^	0.982

**Table 2:** Thermodynamic parameters.

T (K)	K_eq_	Ln (K_eq_)	ΔH° (KJ/mol)	ΔS° (J/mol∙K)	ΔG°_278_ (KJ/mol)
278	70	4.25	+(49.8 ± 4.0)	+(214.2 ± 14.0)	−(9.7 ± 7.8)
288	135	4.90			−(11.8 ± 8.0)
298	300	5.70			−(14.0 ± 8.2)

**Table 3:** Consecutive cycles of adsorption referring to experiments of adsorption in which the Kp concentration was maintained at 1 × 10^−5^ M, pH 5, and the amount of chitosan was 150 mg.

N° of Cycles	Percentage of Adsorption
1	77 ± 5
2	82 ± 8
3	73 ± 7
4	77 ± 10
5	76 ± 5
6	73 ± 5
7	68 ± 8
8	68 ± 6

**Table 4:** Consecutive cycles of adsorption and desorption (obtained in the presence of 0.25 M NaCl, with a contact time of 30 min) referring to experiments of adsorption in which the Kp concentration was maintained at 1 × 10^−5^ M, pH 5, and the amount of chitosan was 150 mg.

N° of Cycles	Percentage of Adsorption	Percentage of Desorption
1	84 ± 5	95 ± 3
2	84 ± 5	92 ± 7
3	89 ± 5	95 ± 5
4	90 ± 5	95 ± 5
5	89 ± 5	95 ± 5
6	91 ± 5	95 ± 5
7	89 ± 4	95 ± 5
8	85 ± 4	93 ± 6
9	80 ± 4	90 ± 5
10	75 ± 4	90 ± 4

## References

[1] Divya K., Rebello S., Jisha M.S. A Simple and Effective Method for Extraction of High Purity Chitosan from Shrimp Shell Waste. Proceedings of the International Conference on Advances in Applied Science and Environmental Engineering—ASEE 2014.

[2] Teli M.D., Sheikh J. (2012). Extraction of chitosan from shrimp shells waste and application in antibacterial finishing of bamboo rayon. Int. J. Biol. Macromol..

[3] Ahing F.A., Wid N. (2016). Extraction and Characterization of Chitosan from Shrimp Shell Waste in Sabah. Trans. Sci. Technol..

[4] Premasudha P., Vanathi P., Abirami M. (2017). Extraction and Characterization of Chitosan from Crustacean Waste: A Constructive Waste Management Approach. Int. J. Sci. Res..

[5] Yong S.K., Shrivastava M., Srivastava P., Kunhikrishnan A., Bolan N. (2015). Environmental applications of chitosan and its derivatives. Rev. Environ. Contam. Toxicol..

[6] Kanmani P., Aravind J., Kamaraj M., Sureshbabu P., Karthikeyan S. (2017). Environmental applications of chitosan and cellulosic biopolymers: A comprehensive outlook. Bioresour. Technol..

[7] Rizzi V., Longo A., Placido T., Fini P., Gubitosa J., Sibillano T., Giannini C., Semeraro P., Franco E., Ferrandiz M. (2017). A comprehensive investigation of chitosan/dyes blended films for green chemistry applications. J. Appl. Polym. Sci..

[8] Semeraro P., Fini P., D’Addabbo M., Rizzi V., Cosma P. (2017). Removal from wastewater and recycling of azo textile dyes by alginate-chitosan beads. Int. J. Environ. Agric. Biotechnol. (IJEAB).

[9] Rizzi V., Longo A., Fini P., Semeraro P., Cosma P., Franco E., García R., Ferrándiz M., Núñez E., Gabaldón J.A. (2014). Applicative Study (Part I): The Excellent Conditions to Remove in Batch Direct Textile Dyes (Direct Red, Direct Blue and Direct Yellow) from Aqueous Solutions by Adsorption Processes on Low-Cost Chitosan Films under Different Conditions. Adv. Chem. Eng. Sci..

[10] Kandile N.G., Mohamed H.M. (2019). Chitosan nanoparticle hydrogel based sebacoyl moiety with remarkable capability for metal ion removal from aqueous systems. Int. J. Biol. Macromol..

[11] Rizzi V., Fini P., Fanelli F., Placido T., Semeraro P., Sibillano T., Fraix A., Sortino S., Agostiano A., Giannini C. (2016). Molecular interactions, characterization and photoactivity of Chlorophyll a/Chitosan/2-HP-β-Cyclodextrin composite films as functional and active surfaces for ROS production. Food Hydrocoll..

[12] Rizzi V., Fini P., Semeraro P., Cosma P. (2016). Detailed Investigation of ROS arisen from Chlorophyll a/Chitosan based-biofilm. Colloids Surf. B.

[13] Rizzi V., Prasetyanto E.A., Chen P., Gubitosa J., Fini P., Agostiano A., De Cola L., Cosma P. (2019). Amino grafted MCM-41 as highly efficient and reversible ecofriendly adsorbent material for the Direct Blue removal from wastewater. J. Mol. Liq..

[14] Rizzi V., Fiorini F., Lamanna G., Gubitosa J., Prasetyanto E.A., Fini P., Fanelli F., Nacci A., De Cola L., Cosma P. (2018). Polyamidoamine-Based Hydrogel for Removal of Blue and Red Dyes from Wastewater. Adv. Sustain. Syst..

[15] Gomes A.R., Justino C., Rocha-Santos T., Freitas A.C., Duarte A.C., Pereira R. (2017). Review of the ecotoxicological effects of emerging contaminants to soil biota. J. Environ. Sci. Health A Environ. Sci. Eng..

[16] Rizzi V., D’Agostino F., Fini P., Semeraro P., Cosma P. (2017). An interesting environmental friendly cleanup: The excellent potential of olive pomace for disperse blue adsorption/desorption from wastewater. Dyes Pigments.

[17] Jeirani Z., Niu C.H., Soltan J. (2017). Adsorption of emerging pollutants on activated carbon. Rev. Chem. Eng..

[18] Rizzi V., Lacalamita D., Gubitosa J., Fini P., Petrella A., Romita R., Agostiano A., Gabaldón J.A., Fortea Gorbe M.I., Gómez-Morte T. (2019). Removal of tetracycline from polluted water by chitosan-olive pomace adsorbing films. Sci. Total Environ..

[19] Rizzi V., Mongiovì C., Fini P., Petrella A., Semeraro P., Cosma P. (2017). Operational parameters affecting the removal and recycling of direct blue industrial dye from wastewater using bleached oil mill waste as alternative adsorbent material. Int. J. Environ. Agric. Biotechnol. (IJEAB).

[20] Dai Y., Zhang K., Meng X., Li J., Guan X., Sun Q., Sun Y., Wang W., Lin M., Liu M. (2019). New use for spent coffee ground as an adsorbent for tetracycline removal in water. Chemosphere.

[21] Cuklev F., Fick J., Cvijovic M., Kristiansson E., Förlin L., Larsson D.G.J. (2012). Does ketoprofen or diclofenac pose the lowest risk to fish?. J. Hazard. Mater..

[22] Diniz M.S., Salgado R., Pereira V.J., Carvalho G., Oehmen A., Reis M.A.M., Noronha J.P. (2015). Ecotoxicity of ketoprofen, diclofenac, atenolol and their photolysis byproducts in zebrafish (Danio rerio). Sci. Total Environ..

[23] Hasan H.A., Abdullah S.R.S., Al-Attabi A.W.N., Nash D.A.H., Anuar N., Rahman N.A., Titah H.S. (2016). Removal of ibuprofen, ketoprofen, COD and nitrogen compounds from pharmaceutical wastewater using aerobic suspension-sequencing batch reactor (ASSBR). Sep. Purif. Technol..

[24] Jankunaite D., Tichonovas M., Buivydiene D., Radziuniene I., Racys V., Krugly E. (2017). Removal of Diclofenac, Ketoprofen, and Carbamazepine from Simulated Drinking Water by Advanced Oxidation in a Model Reactor. Water Air Soil Pollut..

[25] Nagy Z.M., Molnár M., Fekete-Kertész I., Molnár-Perl I., Fenyvesi É., Gruiz K. (2014). Removal of emerging micropollutants from water using cyclodextrin. Sci. Total Environ..

[26] Madikizela L.M., Zunngu S.S., Mlunguza N.Y., Tavengwa N.T., Mdluli P.S., Chimuka L. (2018). Application of molecularly imprinted polymer designed for the selective extraction of ketoprofen from wastewater. Water SA.

[27] Rizzi V., Romanazzi F., Gubitosa J., Fini P., Romita R., Agostiano A., Petrella A., Cosma P. (2019). Chitosan Film as Eco-Friendly and Recyclable Bio-Adsorbent to Remove/Recover Diclofenac, Ketoprofen, and their Mixture from Wastewater. Biomolecules.

[28] Sekulic M.T., Boskovic N., Milanovic M., Letic N.G., Gligoric E., Papa S. (2019). An insight into the adsorption of three emerging pharmaceutical contaminants on multifunctional carbonous adsorbent: Mechanisms, modelling and metal coadsorption. J. Mol. Liq..

[29] Anbia M., Salehi S. (2012). Removal of acid dyes from aqueous media by adsorption onto amino-functionalized nanoporous silica SBA-3. Dyes Pigments.

[30] Rizzi V., D’Agostino F., Gubitosa J., Fini P., Petrella A., Agostiano A., Semeraro P., Cosma P. (2017). An Alternative Use of Olive Pomace as a Wide-Ranging Bioremediation Strategy to Adsorb and Recover Disperse Orange and Disperse Red Industrial Dyes from Wastewater. Separations.

[31] Antunes M., Esteves V.I., Guégan R., Crespo J.S., Fernandes A.N., Giovanela M. (2012). Removal of diclofenac sodium from aqueous solution by Isabel grape bagasse. Chem. Eng. J..

[32] Lin K.Y.A., Yang H., Lee W.D. (2015). Enhanced removal of diclofenac from water using a zeolitic imidazole framework functionalized with cetyltrimethylammonium bromide (CTAB). RSC Adv..

[33] Chang J.H., Chen C.L., Ellis A.V., Tung C.H. (2012). Studies of Chitosan at Different pH’s in the Removal of Common Chlorinated Organics from Wastewater. Int. J. Appl. Sci. Eng..

[34] Meloun M., Bordovská S., Galla L. (2007). The thermodynamic dissociation constants of four non-steroidal anti-inflammatory drugs by the least-squares nonlinear regression of multiwavelength spectrophotometric pH-titration data. J. Pharm. Biomed. Anal..

[35] Desbrières J., Guibal E. (2018). Chitosan for wastewater treatment. Polym. Int..

[36] Pereira K.A.A., Osório L.R., Silva M.P., Sousa K.S., Da Silva Filho E.C. (2014). Chemical Modification of Chitosan in the Absence of Solvent for Diclofenac Sodium Removal: pH and Kinetics Studies. Mater. Res..

[37] Zhang Y., Shen Z., Dai C., Zhou X. (2014). Removal of selected pharmaceuticals from aqueous solution using magnetic chitosan: Sorption behavior and mechanism. Environ. Sci. Pollut. Res..

[38] Carvalho T.O., Matias A.E.B., Braga L.R., Evangelista S.M., Prado A.G.S. (2011). Calorimetric studies of removal of nonsteroidal anti-inflammatory drugs diclofenac and dipyrone from water. J. Therm. Anal. Calorim..

